# Erbium-Implanted WS_2_ Flakes with Room-Temperature Photon Emission at Telecom Wavelengths

**DOI:** 10.1021/acs.nanolett.5c01620

**Published:** 2025-05-20

**Authors:** Guadalupe García-Arellano, Gabriel I. López Morales, Zav Shotan, Raman Kumar, Ben Murdin, Cyrus E. Dreyer, Carlos A. Meriles

**Affiliations:** † Department of Physics, 14770 CUNY-City College of New York, New York, New York 10031, United States; ‡ CUNY-Graduate Center, New York, New York 10016, United States; § Advanced Technology Institute, 3660 University of Surrey, Guildford GU2 7XH, United Kingdom; ∥ Department of Physics and Astronomy, Stony Brook University, Stony Brook, New York 11794-3800, United States; ⊥ Center for Computational Quantum Physics, Flatiron Institute, 162 Fifth Avenue, New York, New York 10010, United States

**Keywords:** Telecom photon emission, spin qubits, rare-earth ions, two-dimensional materials, tungsten disulfide

## Abstract

Optically addressable spin impurities in crystals along with device engineering provide an attractive route to realizing quantum technologies in the solid state, but reconciling disparate emitter and host material constraints for a given target application is often challenging. Rare-earth ions in two-dimensional (2D) materials could mitigate this problem given the atomic-like transitions of the emitters and the versatile nature of van der Waals systems. Here we combine ion implantation, confocal microscopy, and ab initio calculations to examine the photon emission of Er-doped WS_2_ flakes. Optical spectroscopy reveals narrow, long-lived photoluminescence lines in the telecom band, which we activate after low-temperature thermal annealing. Spectroscopic and polarization-selective measurements show a uniform response across the ensemble, while the fluorescence brightness remains mostly unchanged with temperature, suggesting nonradiative relaxation channels are inefficient. Our results create opportunities for novel solid state devices coupling 2D-hosted, telecom-band emitters to photonic heterostructures separately optimized for photon manipulation.

## Graphical abstract

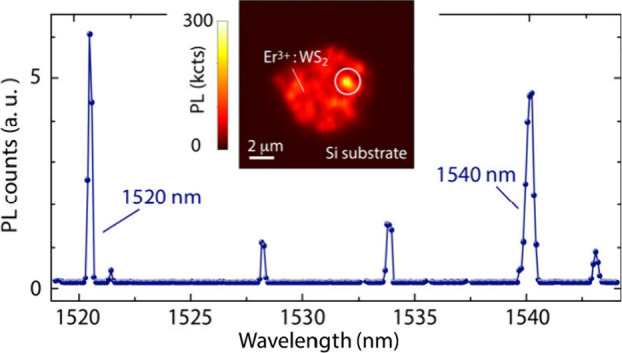

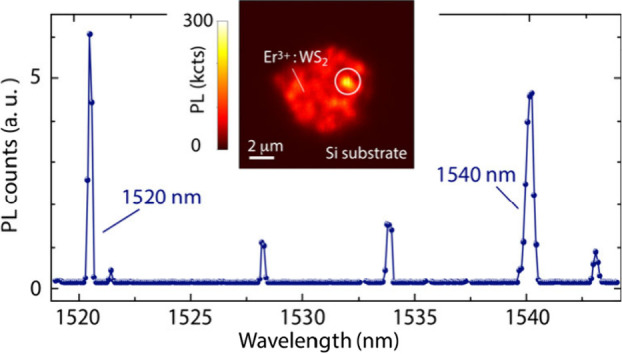

Solid-state quantum registers formed by interacting electron and nuclear spins amenable to high-fidelity state manipulation and readout provide a promising architecture for quantum technologies.^1,2^ Quantum emitters in the form of substitutional rare-earth ions have surfaced as an interesting option because electronic screening of the 4*f* orbitals leads to narrow optical transitions at frequencies relatively insensitive to the crystal host. Since not all materials are equally flexible to device engineering, this feature is advantageous in that it allows the experimenter to separately select the optimal emitter/host system for a given application. With emission in the telecom band,^3^ Er^3+^ ions are especially attractive for long-distance communication and distributed quantum computing, an interest accentuated by recent demonstrations of subdiffraction single-shot readout,^4^ indistinguishable photon generation,^5^ quantum storage of photon states,^6,7^ and spectral multiplexing.^8^

While most applications to quantum information processing have focused thus far on rare-earth ions in garnets or oxides,^9^ an intriguing possibility is the use of two-dimensional (2D) hosts,^10,11^ especially in multilayer form. Unlike monolayers—where emitters are subject to environmental fluctuations difficult to control—these “quasi-2D” hosts provide bulk-like crystalline environments, which facilitates control of the qubit properties through the use of metamaterial structures,^12,13^ local strain,^14–16^ or electric fields.^17–19^ Given the subwavelength distances involved, 2D-hosted emitters can be near-field coupled to photonic structures explicitly designed for photon manipulation.^20^

From a broad palette of 2D systems, tungsten disulfide (WS_2_) garners particular interest given its chemical stability, high electron mobility,^21^ and bandgap tunability.^22^ All nuclear-spin-active isotopes have low natural abundances suggesting Er ions in this material could serve as long-lived spin qubits.^23,24^ Moreover, the nonpolar nature of the crystalline lattice should render substitutional color centers robust to noise processes (e.g., moving charges) known to degrade the emitter’s optical and spin properties, especially close to the surface.^25,26^

Er-doped WS_2_ nanosheets in the form of composite films have recently been examined as a platform for photon up- and down-conversion,^27^ infrared (IR) photodetection,^27^ and photothermal imaging.^28^ In the same vein, chemical vapor deposition (CVD) has been shown to yield large-area, Er:WS_2_ flakes with Er photoluminescence (PL) in the infrared.^29,30^ Unfortunately, all reported spectra feature broad emission lines (50–100 nm) pointing to crystalline disorder and/or contributions from nonequivalent Er ions, inadequate for quantum information processing applications.

Here, we articulate confocal microscopy and density functional theory to study the telecom PL stemming from Er emitters in exfoliated WS_2_ flakes. Variable temperature optical spectroscopy reveals a collection of emission lines featuring subnm inhomogeneous line widths and ms-long lifetimes, as well as a high-degree of linear polarization. Using embedding techniques to properly capture the many-body nature of the ion, we derive emission spectra and polarization plots for substitutional Er^3+^ emitters in qualitative agreement with our observations.

The sample we examine comprises a collection of WS_2_ flakes exfoliated from a high-purity crystal and transferred onto a Si substrate. In the absence of available CVD reactors,^29,30^ we resort to broad-area Er ion implantation. Unlike incorporation during growth, ion bombardment introduces lattice damage, which here proved sufficient to quench all Er fluorescence in the as-implanted flakes (possibly due to high vacancy concentration^29^). We circumvented this problem via a soft, 1-h-long thermal annealing at 400 °C in an Ar environment (see Supporting Information (SI), Section I). These conditions are seemingly sufficient to improve the crystallinity of WS_2_ and selectively activate Er emission in the flakes without also triggering fluorescence from ions in the Si substrate.^31^

We use a custom-made confocal microscope with excitation at 980 nm (Figure 1a) to examine flake sets exposed to varying Er implantation doses (see SI, Section I for details). Figure 1b displays two examples—Flakes 1 and 2, with implantation dose of 10^14^ ions/cm^2^—here imaged optically upon reconfiguring the microscope to monitor the back-reflection of the 980 nm excitation beam. As shown in the fluorescence maps of Figure 1c, only the first one exhibits telecom-band PL. Our observations indicate the flake thickness—smaller for Flake 2, see Figure 1d—is the underlying cause. Indeed, a systematic comparison between flakes shows that a thickness of at least 200 nm is necessary to observe Er fluorescence (SI, Section I). For the present implantation energy (75 keV), we find a penetration depth of ∼400 nm which, in turn, exposes a large discrepancy with predictions for Er ions extracted from SRIM^32^ modeling.^33–36^

**Figure 1:**
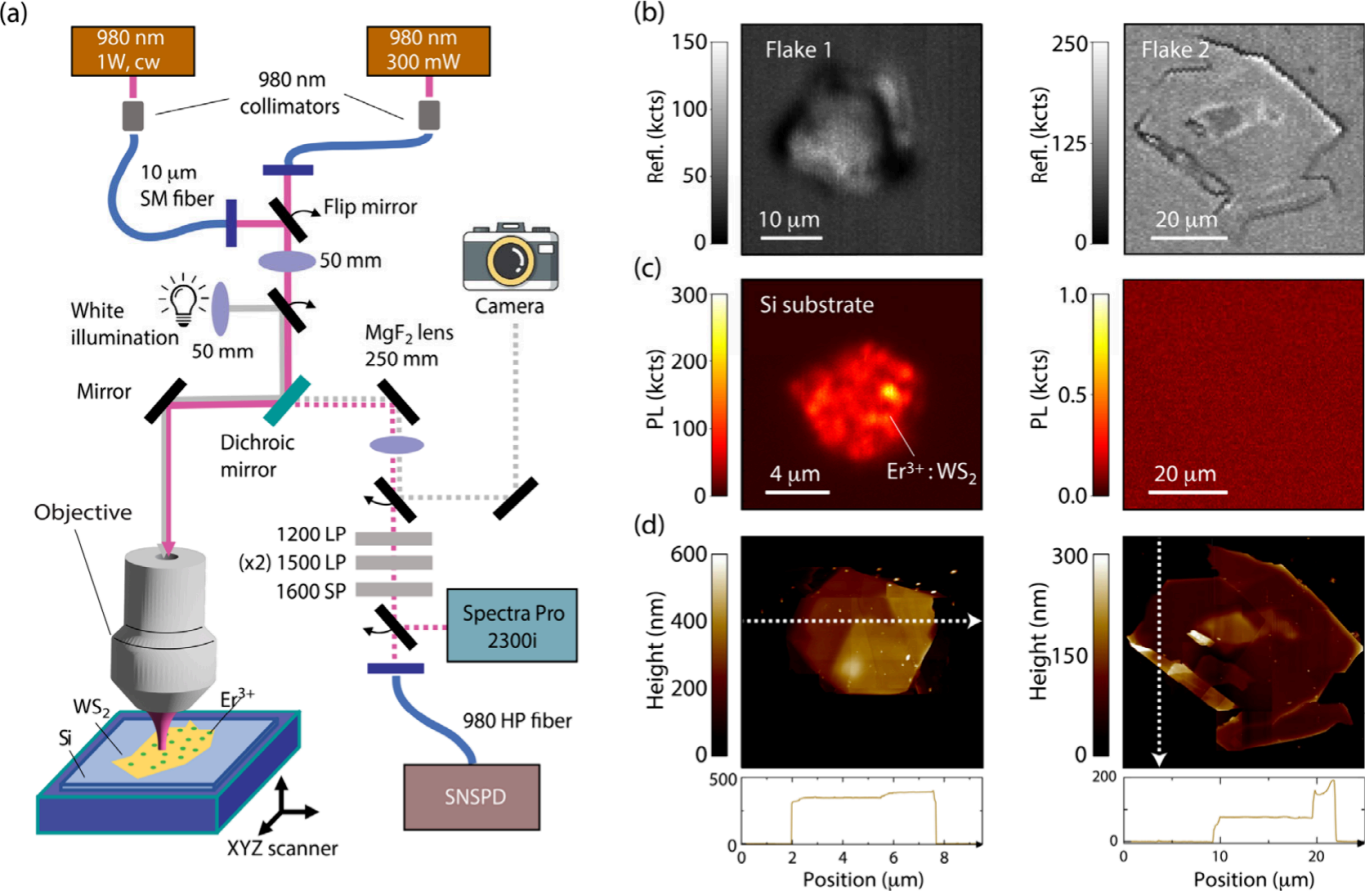
**Infrared fluorescence confocal microscopy of exfoliated WS**_**2**_ **.** (a) Schematic of the experimental setup. Depending on the target experiment, we resort to cw or gated 980 nm lasers for excitation; both lasers are linearly polarized. We use three long-pass filters (one at 1200 nm and two at 1500 nm) as well as a short-pass filter (at 1600 nm) to minimize photon contributions away from the telecom bands. (b) Reflection-mode confocal images of select flakes on the silicon substrate. In this modality, we remove all bandpass filters and collect the excitation beam reflection upon proper attenuation. (c) Confocal fluorescence images of the flakes shown in (b). Flake 2 yields no fluorescence, a consequence of its thickness being thinner than the penetration depth of the Er ions during implantation. (d) Atomic force microscopy of the flakes in (b). The lower plots are cross sections of the images along the dashed white lines. All experiments at room temperature. SNSPD: Superconducting nanowire single photon detector. LP: Long-pass filter. SP: Short-pass filter. SM: Single mode fiber. HP: High power fiber.

To investigate the optical response of the WS_2_-hosted emitters, we now turn our attention to Flake 1 where optical spectroscopy across the 1300–1600 nm range reveals a collection of PL emission peaks featuring subnm inhomogeneous line widths, often limited by the spectrometer resolution (0.1 nm). Qualitatively, we interpret our observations as the result of a cascade process, first involving relaxation from ^4^I_11/2_ (pumped via 980 nm excitation) into ^4^I_13/2_, followed by photon emission upon decay to the ground state ^4^I_15/2_ (Figure 2a); exchange and crystal field (CF) interactions transform into manifolds each of these high-multiplicity levels, hence leading to several emission lines, as observed.

**Figure 2:**
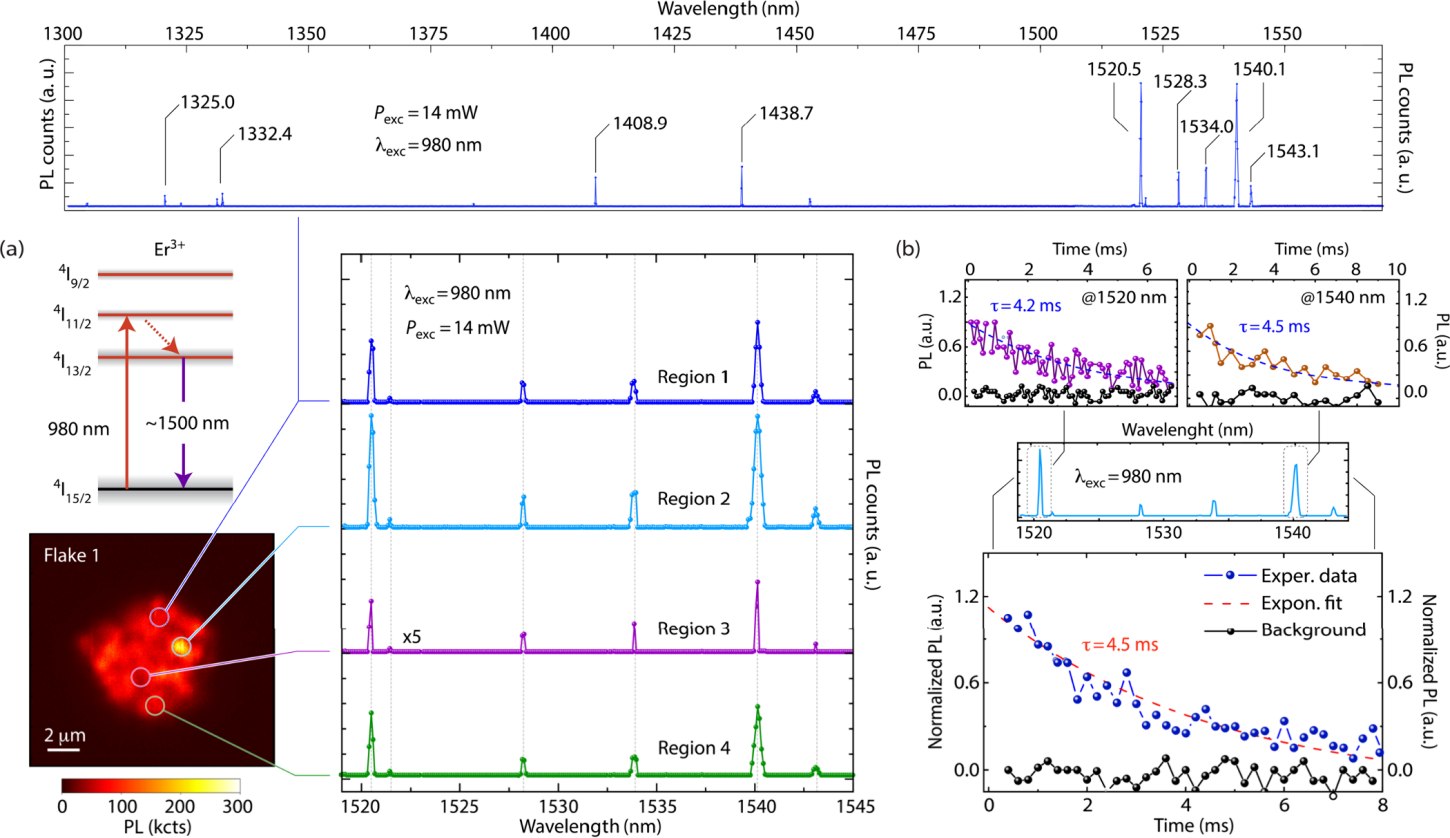
**Optical characterization of Er-implanted WS**_**2**_ **flakes.** (a) Schematic energy diagram (top left) and fluorescence confocal image of Flake 1 (bottom left, reproduced from Figure 1 for clarity). Colored circles highlight the areas of the flake corresponding to the optical spectra on the top and right-hand side plots. In all cases, we use 14 mW, 980 nm laser excitation; spectra have been displaced vertically for clarity. (b) (Bottom) PL amplitude integrated over a 1500–1600 nm window (blue dots) as a function of the time elapsed after pulsed optical excitation. From an exponential fit (red, dashed line), we derive an excited state lifetime τ = 4.5 ms. Black dots indicate the observed response in the absence of an excitation pulse. (Upper inserts) Same as before but for selective detection at 1520 and 1540 nm (left and right plots, respectively). All experiments at room temperature.

Spectra from different sites reveal excellent uniformity, suggesting good crystallinity. Note, however, that areas with lower integrated PL intensity tend to exhibit the narrowest lines (see, e.g., spectrum from Region 3 in Figure 2a), which hints at some residual lattice heterogeneity in sections of the crystal with the highest brightness. Flakes exposed to lower Er implantation doses show comparatively more uniform PL maps (as well as improved conversion efficiency upon annealing), possibly because lower ion concentrations prevent Er clustering^37,38^ (SI, Section I).

Optical relaxation in rare-earth ions is typically slow given the shielding of the 4*f* orbitals by lower-energy, outer-radii 6*s* and 5*p* electrons. We confirm this feature in Figure 2b where we monitor the flake’s luminescence as it decays following pulsed optical excitation at 980 nm. We measure a characteristic lifetime τ = 4.5 ± 0.3 ms, comparable to that seen in other high-quality hosts^9^ (SI, Section I). In principle, nonradiative processes could be present although the slow PL decay we measure seems to indicate radiative mechanisms dominate spontaneous relaxation. Observations at temperatures down to 3.5 K—showing little PL change in brightness, frequency, or lifetime, see SI, Section I—confirm this idea.

While optical spectroscopy alone is insufficient to unveil the microscopic structure of the emitters, a substitutional Er_W_ geometry—where Er occupies the site of a W atom in the WS_2_ lattice—seems the most plausible given the much smaller atomic radius of S. In fact, preceding density functional theory (DFT) studies indicated Er_W_ forms a stable point defect with minimum associated lattice distortion.^23,24,29^ Formation energies were seen to favor the neutral and negatively charged defect states, though the electronic occupation of Er remained that of its trivalent form across all charge states (excess electrons occupy defect states stemming from dangling bonds). Further, reduced hybridization of the Er^3+^ orbitals with the WS_2_ lattice resulted in a set of atom-like transitions in the telecom range,^23,24^ although their accuracy is questionable since DFT alone cannot capture the many-body interactions inherent to rare-earth ions.

To describe the optical properties of this system on a more quantitative footing, we calculate the optical matrix elements between many-body states through a correlated semiempirical model within the framework of quantum embedding^39–42^ (see SI, Section II). We assume the Er defect is in the neutral charge state (Er_W_^0^), though our results are qualitatively similar for the negatively charged defect. We consider two situations: In the first scenario, we assume the lowest CF state of ^4^I_13/2_ is the only one populated, and transitions occur to all ^4^I_15/2_ CF states; the second case is one where transitions occur between all ^4^I_13/2_ and ^4^I_15/2_ CF states (Figure 3a and 3b, respectively). The former describes the situation where carriers in the excited ^4^I_13/2_ manifold fully thermalize before the emission process, whereas the latter is the case where there is some population in all ^4^I_13/2_ states. Likely, our experiments lie somewhere between these two limit cases, depending on the details of the relaxation process from the ^4^I_11/2_ states and nonradiative processes within the dense ^4^I_13/2_ manifold.^9,43–47^ In both cases, we see that Er_W_^0^ indeed features bright emission in a region consistent with that observed, but the number of PL lines changes, hence complicating a comparison with experiment.

**Figure 3:**
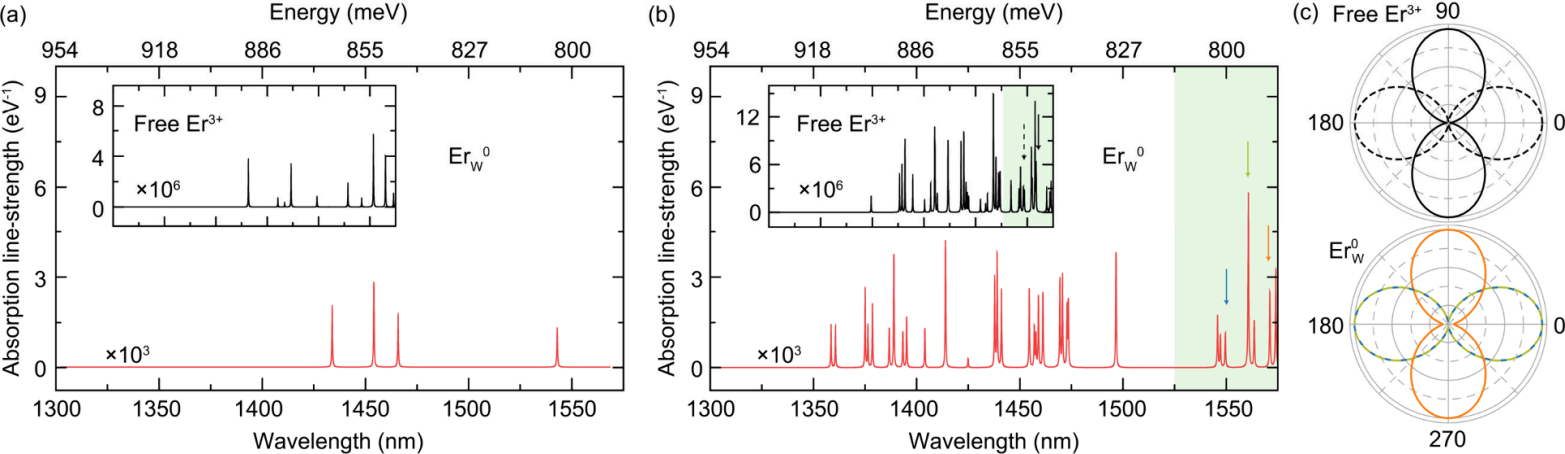
**Calculated** ^**4**^ **I**_**15/2**_ ↔ ^**4**^ **I**_**13/2**_ **optical transitions of Er**_**W**_ **in WS**_**2**_ **.** (a) Absorption line strengths between the lowest-energy crystal-field (CF) state of the ^4^I_15/2_ manifold (ground state) and all ^4^I_13/2_ CF states. The inset compares the same for the case of the isolated Er^3+^ ion in free space. (b) Optical transition strengths from all CF states of the ^4^I_15/2_ manifold to all the CF states of the ^4^I_13/2_ manifold. Green shaded regions represent the region of focus in the optical experiments of Figure 2. All lines were convolved with a 0.1-meV-broad Lorentzian for clarity. (c) Polarization dependence of select CF transitions in (b), as highlighted by color-coded arrows. In the top panel of (c), the polarization of two proximal transitions (black arrows in the inset to (b)) is represented by solid and dashed lines to illustrate different degrees of polarization in the transitions of the free ion. The bottom panel of (c) exemplifies the polarization for three CF transitions of the Er_W_^0^ defect that lie close to 1550 nm (color-coded arrows in (b)); note we use a dashed line for the blue trace so as to reveal the underlying green trace.

Our calculations, however, do provide some valuable insights. For example, in Figures 3a and 3b we plot the results for an isolated Er^3+^ ion in a box with the same dimensions as the defect cell. In this case, the CF splittings derive from unscreened interactions between the charged Er^3+^ ion and its periodic images. The fact that the inter-*f* transitions in the free Er^3+^ are 3 orders of magnitude lower than in Er_W_^0^ shows that hybridization with the crystal, and not just a CF interaction, is necessary to activate Er emission. As a matter of fact, the excitation amplitudes of many of the individual ^4^I_15/2_ ↔ ^4^I_13/2_ CF transitions in Er_W_^0^ result in radiative lifetimes between 3–10 ms, which fall within those measured (Figure 2b).

Comparing the calculated optical matrix elements along orthogonal directions in the cell, we find that most of the CF transitions feature a strong degree of polarization. We illustrate this dependence in Figure 3c where we choose a few transitions near 1540 nm and calculate the angular response of its net transition dipole moment. Note that the strong polarization in the isolated ion suggests this dependence is intrinsic to the 4*f* ↔ 4*f* transitions in Er^3+^.

While establishing a one-to-one correspondence between the calculated and measured transitions seems presently unwarranted, experiment can help validate the predicted polarization dependence. As an illustration, Figure 4 shows data from Flake 3, produced under conditions identical to Flake 1. In agreement with ab initio modeling, selective imaging of the 1540 nm PL peak reveals a strong polarization dependence, both on excitation and emission (Figures 4a and 4b). Further, a comparison with polarization plots at 1521 nm (Figure 4c) shows that the direction of the emission dipole changes by 90 deg relative to that observed at 1540 nm, qualitatively consistent with the ab initio predictions in Figure 3c. Interestingly, we find the excitation and emission dipoles are invariably parallel, even for transitions featuring perpendicular emission polarization (Figure 4c): Given the off-resonant nature of the excitation (see Figure 1a), this observation suggests that ^4^I_11/2_ relaxation into the ^4^I_13/2_ manifold follows polarization-preserving selection rules. As a whole, our observations and ab initio calculations correlate well with previous work^47–50^ (SI, Sections I and II).

**Figure 4:**
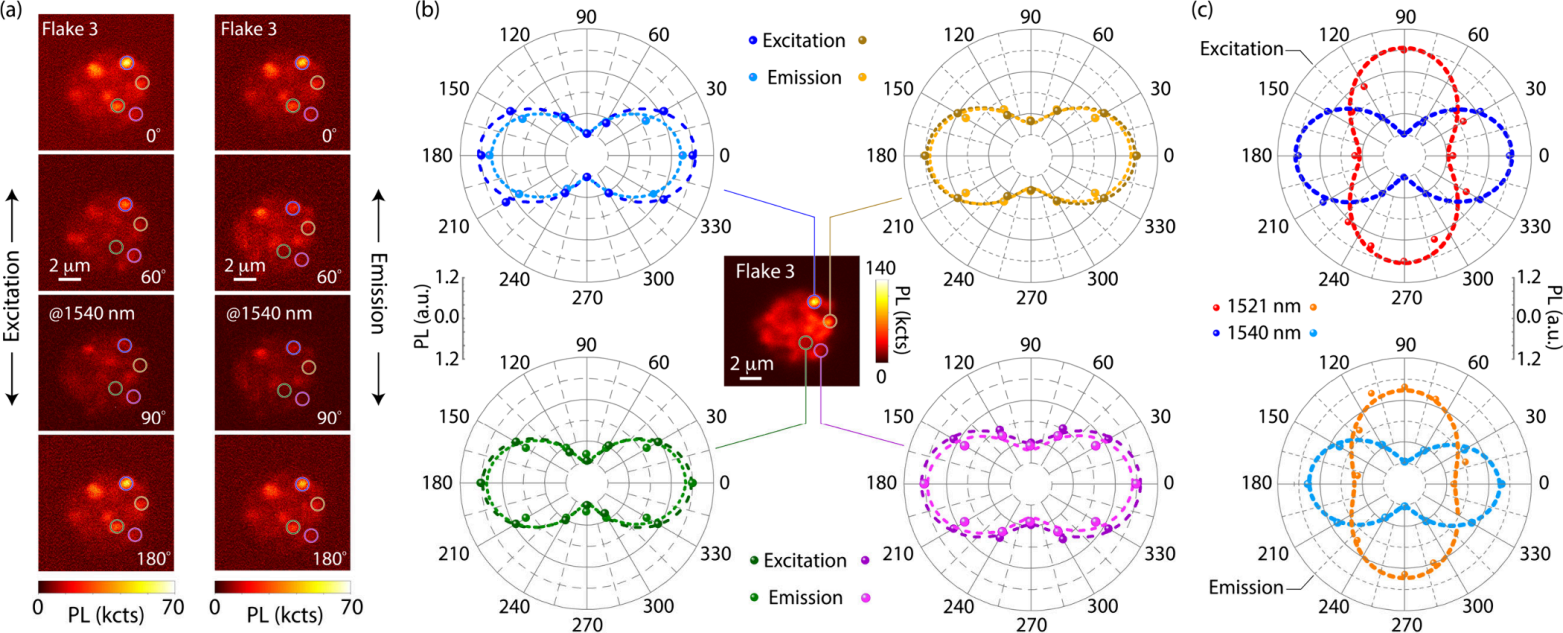
**Polarization dependence of Er** ^**3+**^ **photoluminescence.** (a) PL images from Flake 3 (10^14^ ions/cm^2^ at 75 keV) for varying angle of polarization θ of the excitation beam (left row), or when changing the angle of a linear polarizer prior to PL detection (right row). (b) Excitation and emission polarization plots for four different regions of Flake 3 (colored circles in the PL images); dashed lines in each polar plot are fits to offset sinusoidals. Except the inset image (integrating the PL over the 1500–1600 nm range), all measurements limit photon detection to a 10 nm window centered at 1540 nm. (c) Excitation and emission polarization plots (top and bottom, respectively) at 1521 and 1540 nm; in either case we limit photon collection to 10 nm windows. All observations at room temperature.

In summary, we combined sample engineering and IR microscopy to demonstrate telecom emission of Er^3+^ in exfoliated WS_2_. Optical spectroscopy reveals a pattern of narrow PL lines with minimal heterogeneity, an indication of good crystalline quality. The PL brightness and lifetime depend only weakly on temperature, from cryogenic to ambient conditions, comparing favorably to the behavior of Er in other hosts.^51^ Quantum embedding calculations of Er_W_ show that the presence of the WS_2_ lattice activates nearly forbidden transitions in the Er^3+^ ion core to yield a set of PL lines in the telecom band. Our modeling predicts linearly polarized transition dipoles with transition-dependent, perpendicular orientations, a feature we also observed experimentally.

Our findings suggest a new route to engineering spin-photon interfaces benefiting from the inner-shell transitions of rare-earth emission and the transfer-ready nature of the 2D host. For example, photonic structures may be designed to combine optical resonators and waveguides to simultaneously stimulate telecom emission, efficiently capture outgoing photons, and distribute information to other nodes in a chip; near-field coupling to 2D-crystal-embedded emitters^20^ could therefore alleviate constraints otherwise present if the same host material must fulfill these disparate functions.

Future work must examine the Er^3+^ optical and spin coherences as this is an essential element of a spin-photon interface, in turn, critical in applications where the ion serves as a spin qubit or for microwave-to-optical photon transduction.^52^ The low natural abundance of all nuclear spin active isotopes portends long spin coherence lifetimes provided the concentration of coexisting paramagnetic impurities can be kept in check. By the same token, one expects long-lived optical coherences if indeed the ion sits at a W site (which is nonpolar); since Stark shifts are canceled only to first order, however, high-quality WS_2_ flakes featuring low concentrations of dangling bonds and surface charges will be necessary to bring spectral diffusion to a minimum.

A variety of rare-earth ions with transitions in the visible, infrared, and microwave ranges provide opportunities for extending the present results. Besides Er^3+^, other ions of interest include Yb^3+^ (featuring good spin and optical properties^53,54^ ), Eu^3+^ (seen to attain record spin coherence times^55^), and Ce^3+^ (displaying comparatively high brightness^56^). In the same vein, other 2D hosts are possible including WSe_2_ and WTe_2_, immediate extensions where isotopic dilution can bring down the concentration of spin-active nuclei. Of special note is the study of 2D magnetic hosts such as CrBr_3_,^57^ where rare-earth ions can serve as local reporters.^58^

## Supplementary Material



## Data Availability

The data that support the findings of this study are available from the corresponding author upon reasonable request.

## Notes

Details on the experimental setup, measurement protocols and conditions, characterization of WS_2_ with lower implantation dose, optical spectroscopy and lifetime measurements under cryogenic conditions, and details on ab initio modeling (PDF)

## References

[1] Awschalom D. D., Hanson R., Wrachtrup J., Zhou B. B. (2018). Quantum technologies
with optically interfaced solid-state spins. Nat. Phot..

[2] Atatüre M., Englund D., Vamivakas N., Lee S.-Y., Wrachtrup J. (2018). Material platforms
for spin-based photonic quantum technologies. Nat. Rev. Mater..

[3] Dibos A. M., Raha M., Phenicie C. M., Thompson J. (2018). Atomic source of single
photons in the telecom band. Phys. Rev. Lett..

[4] Chen S., Raha M., Phenicie C. M., Ourari S., Thompson J. D. (2020). Parallel
single-shot measurement and coherent control of solid-state spins
below the diffraction limit. Science.

[5] Ourari S., Dusanowski Ł., Horvath S. P., Uysal M. T., Phenicie C. M., Stevenson P., Raha M., Chen S., Cava R. J., de Leon N. P., Thompson J. D. (2023). Indistinguishable telecom band photons
from a single Er ion in the solid state. Nature.

[6] Saglamyurek E., Jin J., Verma V. B., Shaw M. D., Marsili F., Nam S. W., Oblak D., Tittel W. (2015). Quantum storage of entangled telecom-wavelength
photons in an erbium-doped optical fiber. Nat.
Phot..

[7] Craiciu I., Lei M., Rochman J., Kindem J. M., Bartholomew J. G., Miyazono E., Zhong T., Sinclair N., Faraon A. (2019). Nanophotonic
quantum storage at telecommunication wavelength. Phys. Rev. Appl..

[8] Ulanowski A., Merkel B., Reiserer A. (2022). Spectral multiplexing of telecom
emitters with stable transition frequency. Sci.
Adv..

[9] Stevenson P., Phenicie C. M., Gray I., Horvath S. P., Welinski S., Ferrenti A. M., Ferrier A., Goldner P., Das S., Ramesh R., Cava R. J., de Leon N. P., Thompson J. D. (2022). Erbium-implanted
materials for quantum communication applications. Phys. Rev. B.

[10] Kianinia M., Xu Z.-Q., Toth M., Aharonovich I. (2022). Quantum emitters
in 2D materials: Emitter engineering, photophysics, and integration
in photonic nanostructures. Appl. Phys. Rev..

[11] Azzam S. I., Parto K., Moody G. (2021). Prospects
and challenges of quantum
emitters in 2D materials. Appl. Phys. Lett..

[12] Proscia N. V., Collison R. J., Meriles C. A., Menon V. M. (2019). Coupling of deterministically
activated quantum emitters in hexagonal boron nitride to plasmonic
surface lattice resonances. Nanophot..

[13] Tran T. T., Wang D., Xu Z.-Q., Yang A., Toth M., Odom T. W., Aharonovich I. (2017). Deterministic
coupling of quantum
emitters in 2D materials to plasmonic nanocavity arrays. Nano Lett..

[14] Proscia N., Shotan Z., Jayakumar H., Reddy P., Dollar M., Alkauskas A., Doherty M. W., Meriles C. A., Menon V. M. (2018). Near-deterministic
activation of single-photon emitters in hexagonal boron nitride. Optica.

[15] Branny A., Kumar S., Proux R., Gerardot B. D. (2017). Deterministic strain-induced
arrays of quantum emitters in a two-dimensional semiconductor. Nat. Commun..

[16] Parto K., Azzam S. I., Banerjee K., Moody G. (2021). Defect and strain engineering
of monolayer WSe2 enables site-controlled single-photon emission up
to 150 K. Nat. Commun..

[17] Palacios-Berraquero C., Barbone M., Kara D. M., Chen X., Goykhman I., Yoon D., Ott A. K., Beitner J., Watanabe K., Taniguchi T., Ferrari A. C., Atatüre M. (2016). Atomically
thin quantum light-emitting diodes. Nat. Commun..

[18] Clark G., Schaibley J. R., Ross J., Taniguchi T., Watanabe K., Hendrickson J. R., Mou S., Yao W., Xu X. (2016). Single defect light-emitting diode
in a van der Waals heterostructure. Nano Lett..

[19] Schwarz S., Kozikov A., Withers F., Maguire J., Foster A., Dufferwiel S., Hague L., Makhonin M., Wilson L., Geim A., Novoselov K. S., Tartakovskii A. I. (2016). Electrically
pumped single-defect light emitters in WSe2. 2D Mater..

[20] Proscia N. V., Jayakumar H., Ge X., López Morales G., Shotan Z., Zhou W., Meriles C. A., Menon V. M. (2020). Microcavity-coupled
emitters in 2D hexagonal boron nitride. Nanophot..

[21] Zhang W., Huang Z., Zhang W., Li Y. (2014). Two-dimensional semiconductors
with possible high room temperature mobility. Nano Res..

[22] Bernardi M., Palummo M., Grossman J. C. (2013). Extraordinary
sunlight absorption
and one nanometer thick photovoltaics using two-dimensional monolayer
materials. Nano Lett..

[23] López
Morales G. I., Hampel A., López G. E., Menon V. M., Flick J., Meriles C. A. (2022). Ab-initio investigation
of Er^3+^ defects in tungsten disulfide. Comput. Mater. Sci..

[24] Khan M. A., Leuenberger M. N. (2021). Ab initio calculations for electronic and optical properties
of Er_W_ defects in single-layer tungsten disulfide. J. Appl. Phys..

[25] Raha M., Chen S., Phenicie C. M., Ourari S., Dibos A. M., Thompson J. D. (2020). Optical quantum
nondemolition measurement of a single
rare earth ion qubit. Nat. Commun..

[26] Sangtawesin S., Dwyer B. L., Srinivasan S., Allred J. J., Rodgers L. V. H., De Greve K., Stacey A., Dontschuk N., O’Donnell K. M., Hu D., Evans D. A., Jaye C., Fischer D. A., Markham M. L., Twitchen D. J., Park H., Lukin M. D., de Leon N. P. (2019). Origins of diamond
surface noise
probed by correlating single-spin measurements with surface spectroscopy. Phys. Rev. X.

[27] Li Q., Rao H., Mei H., Zhao Z., Gong W., Camposeo A., Pisignano D., Yang X. (2022). Erbium-doped WS_2_ with
down- and up conversion photoluminescence integrated on silicon for
heterojunction infrared photodetection. Adv.
Mater. Interface.

[28] Huang Y., Zhao Y., Liu Y., Ye R., Chen L., Bai G., Xu S. (2021). Erbium-doped tungsten selenide nanosheets with near-infrared
II emission and photothermal conversion. Chem.
Eng. J..

[29] Zhao H., Zhang G., Yan B., Ning B., Wang C., Zhao Y., Shi X. (2022). Substantially
enhanced properties
of 2D WS_2_ by high concentration of erbium doping against
tungsten vacancy formation. Research.

[30] Wang L., Zhang S., Yan L., Ji X., Zhang Q. (2023). Chemical vapor
deposition of Er-doped WS_2_ flakes with near-infrared emission
and enhanced near-infrared photoresponse. J.
Phys. Chem. C.

[31] Gritsch L., Weiss L., Früh J., Rinner S., Reiserer A. (2022). Narrow optical
transitions in erbium-implanted silicon waveguides. Phys. Rev. X.

[32] Ziegler J. F., Ziegler M. D., Biersack J. P. (2010). ″SRIM - The stopping and range
of ions in matter″. Nucl. Instr. Meth.
Phys. Res. B.

[33] Eder K., Bhatia V., Qu J., Van Leer B., Dutka M., Cairney J. M. (2021). A multi-ion plasma
FIB study: Determining ion implantation
depths of Xe, N, O and Ar in tungsten via atom probe tomography. Ultramicroscopy.

[34] Moll S., Zhang Y., Zhu Z., Edmondson P. D., Namavar F., Weber W. J. (2013). Comparison between simulated and
experimental Au-ion profiles implanted in nanocrystalline ceria. Nucl. Instr. Meth. Phys. Res. B.

[35] Wittmaack K. (2004). Reliability
of a popular simulation code for predicting sputtering yields of solids
and ranges of low-energy ions. J. Appl. Phys..

[36] Zhang Y., Bae I.-T., Sun K., Wang C., Ishimaru M., Zhu Z., Jiang W., Weber W. J. (2009). Damage profile and ion distribution
of slow heavy ions in compounds. J. Appl. Phys..

[37] Kucheyev S. O., Bradby J. E., Ruffell S., Li C. P., Felter T. E., Hamza A. V. (2007). Segregation and
precipitation of Er in Ge. Appl. Phys. Lett..

[38] Prtljaga N., Navarro-Urrios D., Tengattini A., Anopchenko A., Ramírez J. M., Rebled J. M., Estradé S., Colonna J.-P., Fedeli J.-M., Garrido B., Pavesi L. (2012). Limit to the
erbium ions emission in silicon-rich oxide films by erbium ion clustering. Opt. Mater. Exp..

[39] Bockstedte M., Schütz F., Garratt T., Ivády V., Gali A. (2018). Ab initio description of highly correlated states in defects for
realizing quantum bits. npj Quant. Mater..

[40] Ma H., Sheng N., Govoni M., Galli G. (2021). Quantum embedding theory
for strongly correlated states in materials. J. Chem. Theory Comp..

[41] Muechler L., Badrtdinov D. I., Hampel A., Cano J., Rösner M., Dreyer C. E. (2022). Quantum embedding meth- ods for correlated excited
states of point defects: Case studies and challenges. Phys. Rev. B.

[42] López-Morales G. I., Zajac J. M., Flick J., Meriles C. A., Dreyer C. E. (2024). Quantum
embedding study of strain and charge induced stark effects on the
NV– center in diamond. Phys. Rev. B.

[43] Wei T., Tian Y., Tian C., Jing X., Zhang J., Zhang L., Xu S. (2014). Optical spectroscopy
and population
behavior between ^4^I_11/2_ and ^4^I_13/2_ levels of erbium doped germanate glass. Opt. Mater. Express.

[44] Böttger T., Thiel C. W., Sun Y., Cone R. L. (2006). Optical decoherence
and spectral diffusion at 1.5 μm in Er^3+^:Y_2_SiO_5_ versus magnetic field, temperature, and Er^3+^ concentration. Phys. Rev. B.

[45] Yin C., Rancic M., de Boo G. G., Stavrias N., McCallum J. C., Sellars M. J., Rogge S. (2013). Optical addressing
of an individual
erbium ion in silicon. Nature.

[46] Wei T., Tian Y., Chen F., Cai M., Zhang J., Jing X., Wang F., Zhang Q., Xu S. (2014). Mid-infrared
fluorescence, energy transfer process and rate equation analysis in
Er^3+^ doped germanate glass. Sci.
Rep..

[47] Zhang L., Basyrova L., Loiko P., Camy P., Lin Z., Zhang G., Slimi S., Soĺe R. M., Mateos X., Aguiló M., Díaz F., Dunina E., Kornienko A., Griebner U., Petrov V., Wang L., Chen W. (2022). Growth, structure,
and polarized
spectroscopy of monoclinic Er^3+^:MgWO_4_ crystal. Opt. Mater. Express.

[48] Rokhmin A.
S., Nikonorov N. V., Przhevuskii A. K., Chukharev A. V., Ul’yashenko A. M. (2004). Study of
polarized luminescence in
erbium-doped laser glasses. Opt. Spectrosc..

[49] Martínez-Martínez L. O., Hernández-Hernández E., Stepanov S. (2013). Polarization of the
fluorescence excited in erbium-doped fibers in 1490–1570 nm
spectral range. Opt. Commun..

[50] Rokhmin A., Aseev V., Nikonorov N. (2015). Polarized
luminescence of erbium
and thulium ions in glasses. Opt. Mater..

[51] Zhang J., Grant G. D., Masiulionis I., Solomon M. T., Marcks J. C., Bindra J. K., Niklas J., Dibos A. M., Poluektov O. G., Heremans F. J., Guha S., Awschalom D. D. (2024). Optical
and spin coherence of Er spin qubits in epitaxial cerium dioxide on
silicon. npj Quant. Inf..

[52] Bartholomew J. G., Rochman J., Xie T., Kindem J. M., Ruskuc A., Craiciu I., Lei M., Faraon A. (2020). On-chip coherent
microwave-to-optical
transduction mediated by ytterbium in YVO4. Nat. Commun..

[53] Kindem J. M., Ruskuc A., Bartholomew J. G., Rochman J., Huan Y. Q., Faraon A. (2020). Control and single-shot
readout of an ion embedded
in a nanophotonic cavity,. Nature.

[54] Wu C.-J., Riedel D., Ruskuc A., Zhong D., Kwon H., Faraon A. (2023). Near-infrared hybrid
quantum photonic interface for ^171^Yb^3+^ solid-state
qubits. Phys. Rev. Appl..

[55] Serrano D., Karlsson J., Fossati A., Ferrier A., Goldner P. (2018). All-optical
control of long-lived nuclear spins in rare-earth doped nanoparticles. Nat. Commun..

[56] Siyushev P., Xia K., Reuter R., Jamali M., Zhao N., Yang N., Duan C., Kukharchyk N., Wieck A. D., Kolesov R., Wrachtrup J. (2014). Coherent properties
of single rare-earth spin qubits. Nat. Commun..

[57] Snoeren T. J., Pressler K., Kluherz K. T., Walsh K. M., De Yoreo J. J., Gamelin D. R. (2023). Luminescence and
covalency in Ytterbium-doped CrX3
(X = Cl, Br, I) van der Waals compounds. J.
Am. Chem. Soc..

[58] Snoeren T.J., Pressler K., Gamelin D.R. (2024). Optically
resolved exchange splittings
in the doped van der Waals ferromagnet CrBr3:Yb^3+^. Phys. Rev. Materials.

