# Supplementary material for: Erbium-Implanted WS2 Flakes with Room-Temperature Photon Emission at Telecom Wavelengths
Source: Nano Lett. 2025 May 20;25(22):9070–6. doi: 10.1021/acs.nanolett.5c01620 (PMC12142666; doi:10.1021/acs.nanolett.5c01620)
Supplement: Supplementary file 1 [file nl5c01620_si_001.pdf]

Supplementary Information for

Erbium-implanted WS_2 flakes with room-temperature photon emission at telecom wavelengths

Guadalupe García-Arellano[1], Gabriel I. López Morales[1,4], Zav Shotan[1], Raman Kumar[1], Ben Murdin[3], Cyrus E. Dreyer[4,5], and Carlos A. Meriles[1,2,*]

[1]*Department of Physics, CUNY-City College of New York, New York, NY 10031, USA.*
[2]*CUNY-Graduate Center, New York, NY 10016, USA.*
[3]*Advanced Technology Institute, University of Surrey, Guildford GU2 7XH, United Kingdom.*
[4]*Department of Physics and Astronomy, Stony Brook University, Stony Brook, New York, 11794-3800, USA.*
[5]*Center for Computational Quantum Physics, Flatiron Institute, 162 5th Avenue, New York, New York 10010, USA.*

I. Experimental methods
a) Infrared confocal microscope
b) Sample fabrication.
c) Relation between photoluminescence and flake thickness
d) PL measurements in flakes exposed to varying Er ion doses
e) Optical spectroscopy measurements
f) Optical lifetime measurements
g) Polarization measurements

II. Computational methods
a) Quantum Embedding
b) DFT, correlated active space, and computational details

I. Experimental Methods

a) Infrared confocal microscope

Our home-built scanning confocal microscope system features 980-nm excitation lasers and long pass filters to detect emission of Er^{3+} in WS_2. The complete system is depicted in Fig.1a of the main text: The key components include two 980-nm excitation lasers, namely, one 1W laser (Optoengine) operating in continuous wave (cw) mode and a 300-mW pulsed laser diode (Thorlabs, HL63163DG) controlled by an IC Haus EVAL HB driver. Each laser is coupled into a single-mode fiber with a 10-µm core size and then collimated with a 50 mm lens. The scanning system uses an XYZ scanning stage (piezo stage Npoint) and an Olympus objective (NA = 0.8, 50x) mounted on a one-axis piezo stage. The beam spot diameter is around 1 micron. We first use incoherent light to localize flakes on the sample using a camera AMScope aligned parallel to the collection path, then we scan a 980-nm beam across a $(100\ \mu m)^2$ plane with laser power of 14 mW at a dwell time of 10 ms chosen to attain optimal PL counts. Emission from the samples is collected using the reverse optical path and subsequently separated from the excitation light by a dichroic mirror (Thorlabs DMLP-1138). The PL emission is then focused using a 250-mm magnesium fluoride lens into a single-mode fiber (980 HP, 3.6 µm) and directed into a superconducting nanowire single photon detector (SNSPD 980 IDQ Quantique) optimized to detect telecom wavelengths. We use three filters to prevent excitation laser leakage: one 1200-nm low-pass (LP) filter and two-1500 nm LP filters. Photon counts are recorded using a National Instruments card (NI-PCIe 6321) and a home-made LabVIEW program. Experiments conducted at low temperatures utilize a separate — though similar — confocal microscope [i]. For erbium-based experiments, the system is equipped with two 980 nm excitation lasers: a 100 mW CW laser (MDL III 980 100 mW) and a 300 mW pulsed laser diode (Thorlabs, HL63163DG), driven by an IC Haus EVAL HB. The setup includes a Montana CryoWorkstation (CryoAdvance 100) housing a stack of Attocube positioners (two ANPx101 and one ANPz101) for sample manipulation, and a Zeiss 100x objective with NA 0.90; the same SNSPD is used for telecom photon detection. AFM measurements are performed using a *FastScan* atomic force microscope (AFM) from Bruker.

b) Sample fabrication

WS_2 flakes mechanically exfoliated from a high-purity bulk crystal (2D Semiconductors) were transferred onto a 0.5 mm thick silicon substrate ($4 \times 4\ mm^2$). Prior to exfoliation the silicon piece was patterned using UV photolithography to facilitate flake location. As an illustration, Fig. S1 shows an optical image of one region of the sample (0.4 mm x 0.4 mm) where some WS_2 flakes are visible.

The samples were broad-area implanted at 75 keV with doses of 10^{12}, 10^{13}, and 10^{14} ions/cm^2 at an angle of 22 deg to avoid channeling. ***We respectively denote each flake set as B8, B9, and B10***; all results in the main text correspond to the B10 set but below we present results from the other two sets. Following implantation, all samples were annealed at 400°C in an argon atmosphere for 1 hour (Fig. S1b); the temperature was increased at a rate of 6.3 °C/ minute in a tube furnace (Across International, STF1200).

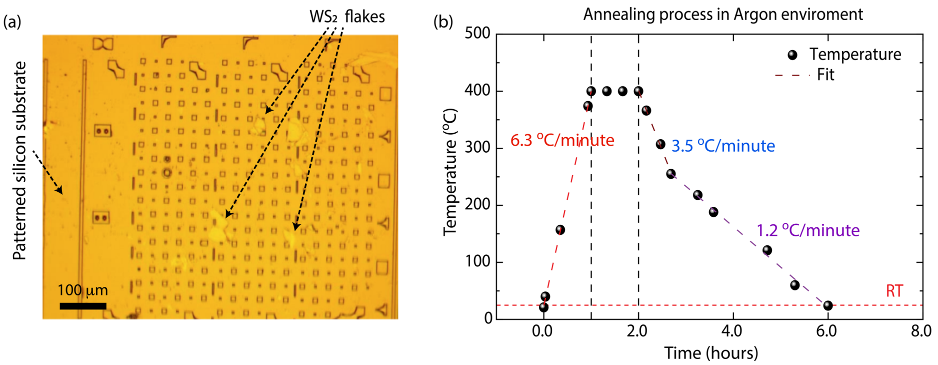

Fig. S1: (a) Optical image of a sample region showing various flakes transferred onto a silicon substrate. (b) Annealing protocol

c) Relation between photoluminescence and flake thickness

Adding to those introduced in the main text, below we present optical, confocal, and AFM images of alternate flakes (Fig. S2). Consistent with the observations in Fig. 1 of the main text, emission can be detected only in regions where the flake thickness is greater than 250 nm.

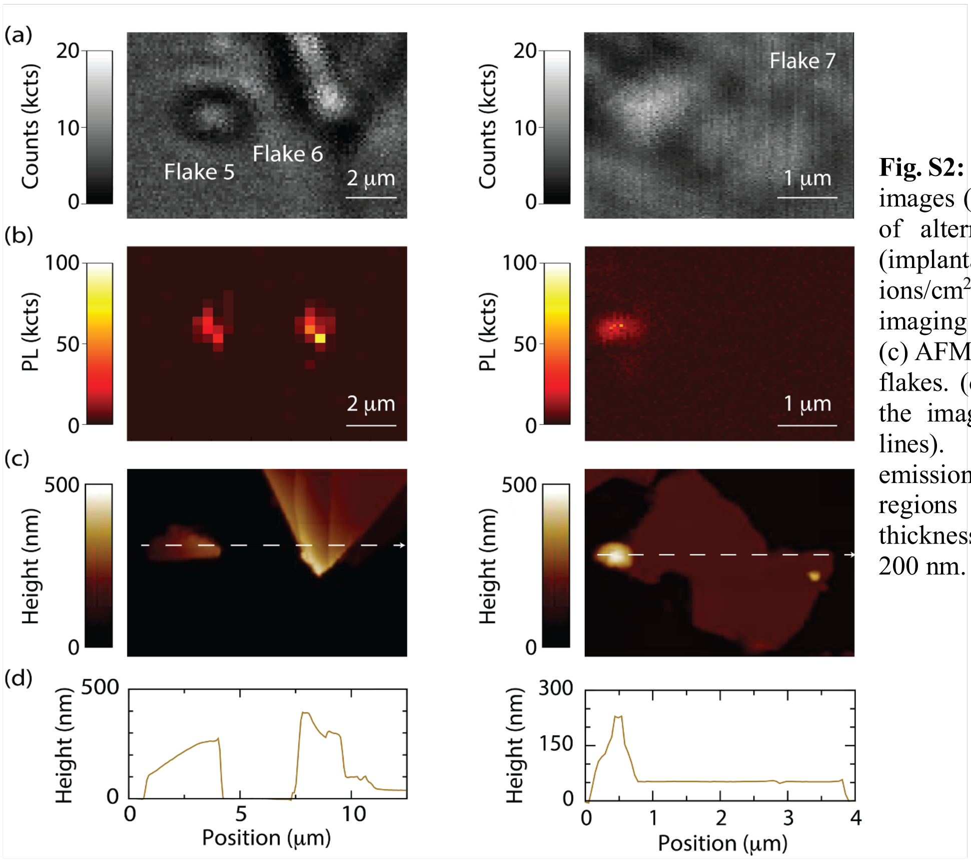

Fig. S2: (a) Confocal optical images (in reflection mode) of alternative B10 flakes (implantation dose of 10^{14} ions/cm^2). (b) Telecom PL imaging for the flakes in (a). (c) AFM images of the same flakes. (d) Cross section of the images in (c) (dashed lines). Notice that PL emission can be seen only in regions where the flake thickness reaches or exceeds 200 nm.

SRIM (Stopping and Range of Ions in Matter) is the traditional software to model ion implantation in bulk materials. However, experimental measurements have shown that it significantly underestimates the penetration depth for ions with atomic number $29 < Z < 83$ implanted in thin target materials, mainly due to an incorrect calculation of the electronic stopping powers and the neglect of channeling effects [1-4]. The

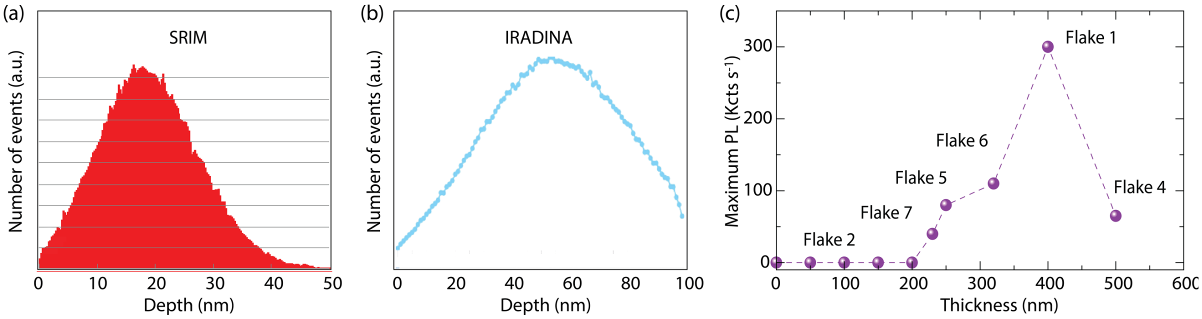

Fig. S3: Modeling of the penetration depth of erbium atoms at 75 keV in WS_2 calculated by (a) SRIM and (b) IRADINA. (c) Thickness vs maximum luminescence detected in different regions of the erbium implanted WS_2 flakes in set B10 (implantation dose of 10^{14} ions cm^{-2}).

factor of underestimation can range from 2 (as reported for erbium-implanted silicon films[5]) to 10 (as reported in Xe, Ar, N, and O ions implanted in tungsten[6]).

Alternative software based on a binary collision approximation (BCA) and a Monte Carlo (MC) transport algorithm have been developed to estimate with better accuracy the penetration depth; examples are IRADINA[7] or TRI3DYN [8]. In Fig. S3a and S3b we show the penetration depth estimated by different software packages in the case of erbium ions implanted at 75 keV in WS_2 at a dose of 10^{14} ions/cm^2: In the case of SRIM the penetration depth estimated is 25 nm whereas IRADINA predicts 60 nm.

In Figure S3c we plot the maximum PL detected in the B10 flakes and their corresponding thicknesses. We find that flakes thicker than 250 nm exhibit telecom luminescence, indicating that the mean penetration depth of the erbium ions for 75 keV must be comparable to 400 nm. We note that ions of this energy may possibly penetrate even deeper, as the crystallinity of exfoliated flakes deteriorates with increasing thickness, potentially affecting the Er ion fluorescence prematurely (likely the case of Flake 2, in Fig. 1).

d) PL measurements in flakes exposed to varying Er ion doses

Besides the B10 set (implantation dose of 10^{14} ions/cm^2), we also examined other flake sets exposed to a lower ion dose. Figures S4a and S4b present observations in select flakes from sets B9 and B8 (implantation doses of 10^{13} and 10^{12} ions/cm^2, respectively); as in the B10 case, we observe telecom emission in cases where the flake thickness is greater than ~200 nm, confirming the conclusion that the penetration depth of 75 keV ions largely exceeds the SRIM estimates. In some occasions, however, we find non-uniformities in the flake brighteness that do not correlate with changes in the flake topography. Examples are the flakes in the first rows of Figs. 4a and 4b where we observe non-fluorescing sections in the PL images that we cannot presently explain; additional work will be needed to clarify this observation.

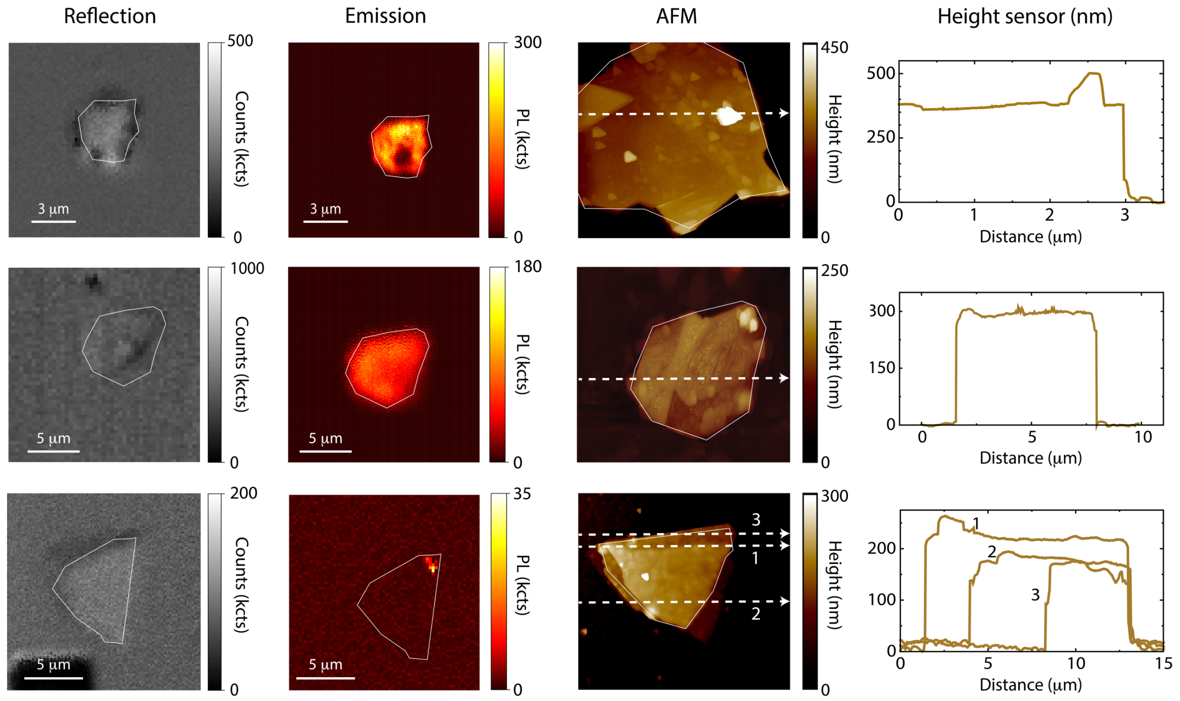

Fig. S4a: Optical, confocal, and AFM images (respectively, first, second, and third columns from the left) of select B9 flakes (10^{13} ions/cm^2 dose). Plots in the last column show the flake topography across the white dashed line in the corresponding AFM image. As in the B10 set (implantation dose of 10^{14} ions/cm^2), we see fluorescence in areas where the flake thickness reaches or exceeds 200 nm. All measurements at room temperature. We used a white contour as a guide to the eye. The AFM image in the second row is a composite from partial images of the same flake.

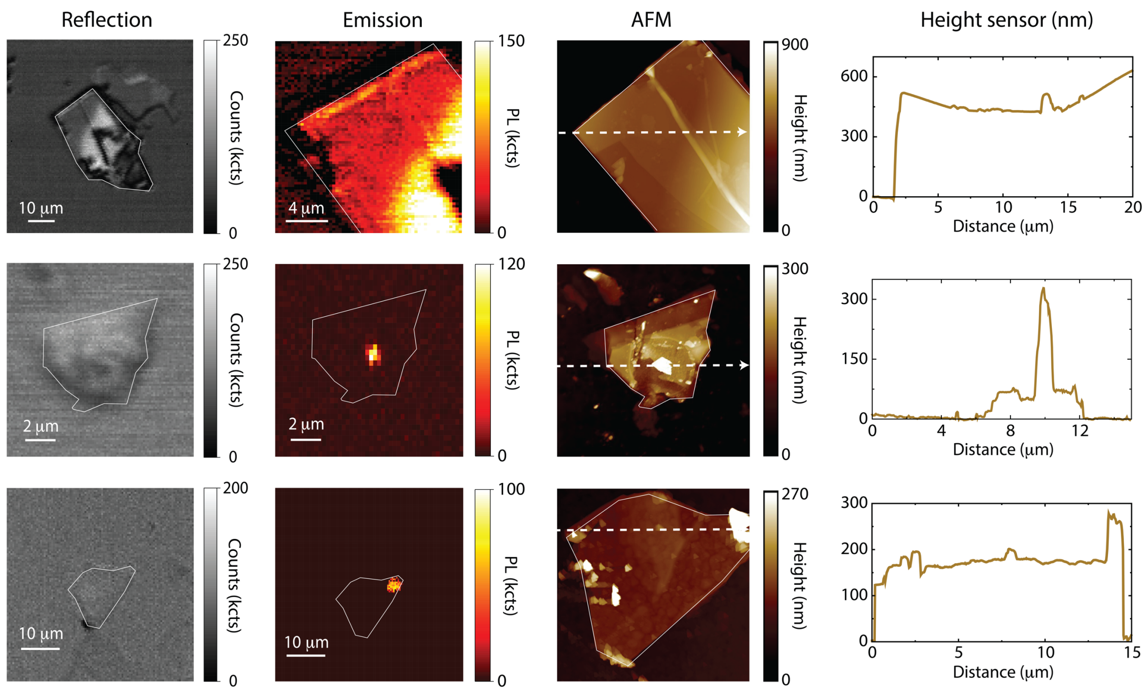

Fig. S4b: Same as in Fig. S4a but for some example flakes in the B8 set (implantation dose of 10^{12} ions/cm^2).

Interestingly, the flakes that do exhibit PL are relatively brighter after accounting for the lower Er content. This is shown in Fig. S5 where we first plot the maximum PL brightness as a function of the WS_2 thickness; crucially, we find in all cases that flake sets exposed to a lower Er dose (10^{12} and 10^{13} ions/cm^2 for sets B8 and B9, respectively) show a PL comparable to that observed in the B10 set (featuring the highest 10^{14} ions/cm^2 implantation dose). This observation suggests that the conversion efficiency into Er^{3+} is significantly higher for lower Er concentration, although the causes remain unclear: Er has been seen to form large precipitates[9,10] for doses of $4.4{\times}10^{15}$ ions/cm^2, a process that could still be at play in the B10 set if annealing can only partially counter atomic aggregation. Consistent with this picture, flakes from the B9 set tend to show a comparatively more uniform brightness (see PL image on the second row of Fig. S4), but additional work will be needed to clarify this point. For completeness, we mention that space charge fields during the implant could potentially lower the ion concentration at the flakes; though possible, this scenario is less likely because it should presumably lead to brightness profiles weaker toward the flake edges, a signature we have not observed.

While determining the ion conversion efficiency necessarily suffers from a large uncertainty, we can leverage the emission rates we measure (see Fig. 2b and below) and the observed PL count at saturation to derive crude estimates of the active Er emitter areal density for each flake set. We derive these values upon

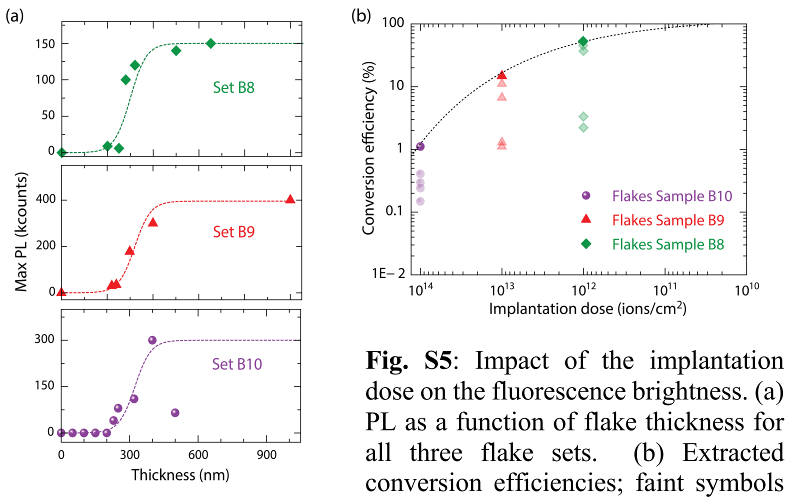

Fig. S5: Impact of the implantation dose on the fluorescence brightness. (a) PL as a function of flake thickness for all three flake sets. (b) Extracted conversion efficiencies; faint symbols represent values derived from flakes with suboptimal thickness as presented in (a). The dashed black line is a guide to the eye.

considering the collection efficiency of our microscope (detecting approximately 3.5% of the total PL) and the area illuminated by the focused laser (~1 μm^2 for our diffraction-limited microscope). Figure S5b shows the results: The conversion efficiency is only 1% for the highest implantation dose (set B10), but grows to reach nearly 30% at lower doses (set B8). We note this value could still improve for optimized annealing recipes, for example, by resorting to slower thermal ramps and/or higher end temperatures.

e) Optical spectroscopy measurements

Our optical spectroscopy measurements use a Spectra Pro 2300i spectrometer from Princeton Instruments equipped with an InGaAs detector and a 1600 nm grating (600 g/mm blaze) optimized to detect 1.5 μm wavelengths. In our experiments, we first localize the luminescent flake using the confocal microscope, and then park the laser beam in the area of choice. The optical signal is then collimated and sent into the spectrometer. The acquisition time of each spectrum is 60 minutes.

Figure S6 extends the spectroscopy measurements presented in the main text to lower temperatures, down to 3.5 K. Qualitatively, we find the emission spectrum remains largely unchanged, both in terms of the characteristic emission frequencies and relative amplitudes (Fig. S6a), suggesting the coupling with the phonon bath is weak. We do observe, however, some narrowing of the inhomogeneous linewidth, from about 40 to 20 GHz (Fig. S6b). Stemming from the brightest site in Flake 1 of set B10 (Region 2 in Fig. 2 in the main text), these linewidths must be seen as an upper bound. Indeed, measurements in the dimmer sections of the flake (e.g., Region 3) show the room temperature emission linewidth (~12 GHz) is comparable to the spectrometer resolution; notably, no spectra from these regions could be recorded under cryogenic conditions, pointing to linewidths below the detection capability of our spectrometer.

The dominant mechanism driving the observed change in inhomogeneous linewidth is presently unknown, but we hypothesize it may originate from strain fluctuations resulting from Raman and direct phonon scattering by the ion[11], or from changes in the local environment arising from thermal expansion of the WS_2 lattice. Using the WS_2 Debye temperature of 213 K as a reference[12] and the mild extra broadening observed at room temperature, we surmise the phonon coupling parameters for Er in WS_2 must be very small. Similar phonon-induced processes influence the values observed for the transition frequencies at a given temperature[11], implying the above conclusion is consistent with the temperature-insensitive resonances observed in Fig. S6. Additional experiments — including the use of a narrow-band tunable laser to implement high-resolution photoluminescence spectroscopy (PLE) — will be helpful in gaining additional insight, for example, through a careful shape analysis of the absorption peaks[11].

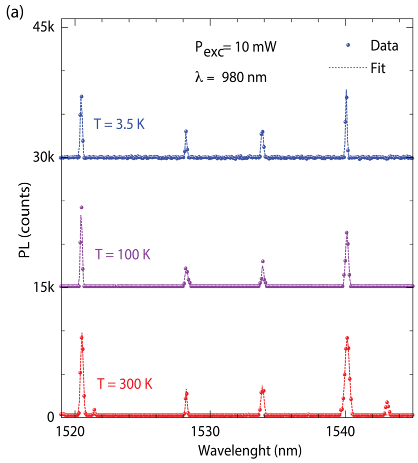

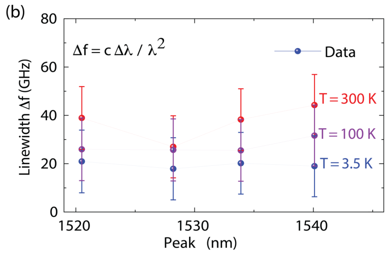

Fig. S6: (a) Optical spectroscopy measurements at different temperatures in Flake 1 recorded at the point of highest brightness (see Fig. 2a in the main text). (b) Inhomogeneous linewidth after a Gaussian fit.

f) Optical lifetime measurements

We use a pulsed 980 nm diode laser operated by an iC Haus Eval HB driver, in turn controlled via a Pulse Blaster from Spincore. We first obtain a confocal image of the bright flake in the sample, then we park the laser beam at the site of choice. Following a 500-μs-long excitation pulse and a variable wait time, we gate the SNSPD to measure the PL over a 200-μs window; we recover the fluorescence decay profile as we sweep the wait time across a 10-ms interval.

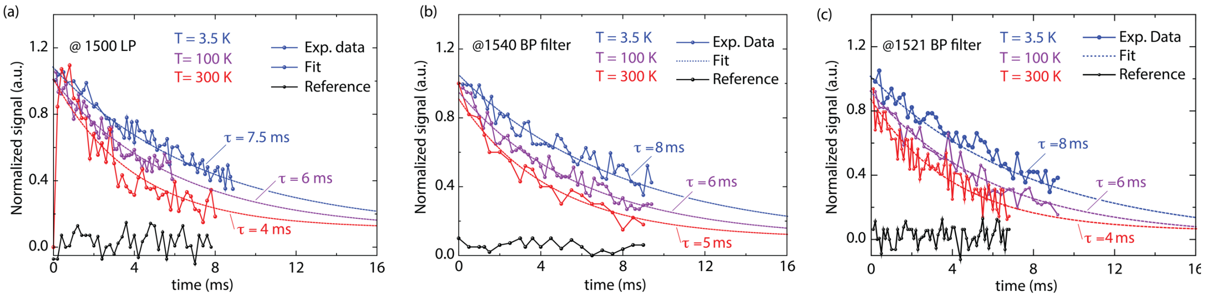

Fig. S7: Measuring the excited state lifetime. (a) Excited state relaxation curve at different temperatures as determined from an integrated PL measurement above 1500 nm. (b, c) Same as in (a) but for PL within a 10-nm window centered at 1540 and 1521 nm, respectively. All measurements were carried out on Flake 1 of the B10 set (see Fig. 1 in the main text). LP: Low pass filter. BP: Bandpass filter.

Figure S7 expands the results in Fig. 2b of the main text to include observations at different temperatures and over different spectral windows, namely broadband detection (1500 – 1600 nm, Fig. S7a) or selectively centered at the 1540 and 1521 nm resonances (Figs. S7b and S7c, respectively). We find nominally the same optical lifetimes τ regardless the spectral window considered, although we do observe a slight increase in τ with decreasing temperature, from ~4 ms at 300 K to ~8 ms at 3.5 K; the latter is likely due to a suppression of non-radiative relaxation channels under cryogenic conditions. The observed lifetimes are comparable to those reported for Er in other hosts including various oxides[13-17], Si[18-27], garnet materials[28], and fluorophosphate glasses[29,30].

g) Polarization measurements

Our experiments integrate a linear polarizer and an achromatic broadband half-wave plate (Thorlabs AHWP05M-980 and AHWP05M-1600) into the excitation and detection paths, respectively. For calibration and to ensure that no artificial effects arise from the optics, we substituted the sample with a fluorescent card (emitting unpolarized light under 980 nm excitation). We then monitored the counts while rotating each of the half-wave plates.

In the excitation path, we also measured the excitation beam power during the rotation of the 980 nm half-wave plate. Emission counts and power data were recorded and are shown in Fig. S8. As illustrated, no systematic variation in either counts or power was observed when rotating the half-wave plates, confirming that the system introduces no artificial polarization effects.

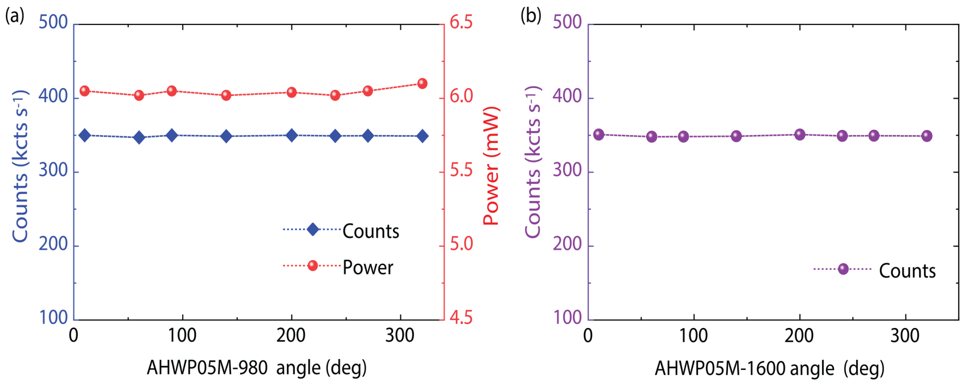

Fig. S8: Control measurements on polarization plates. (a) Laser power (red) and fluorescence counts from an unpolarized source (blue) as we change the orientation of a 980-nm half-wave plate controlling the polarization angle of the 980-nm excitation laser beam. (b) Same as in (a) but for the case where we change the orientation of the half-wave plate in the detection path.

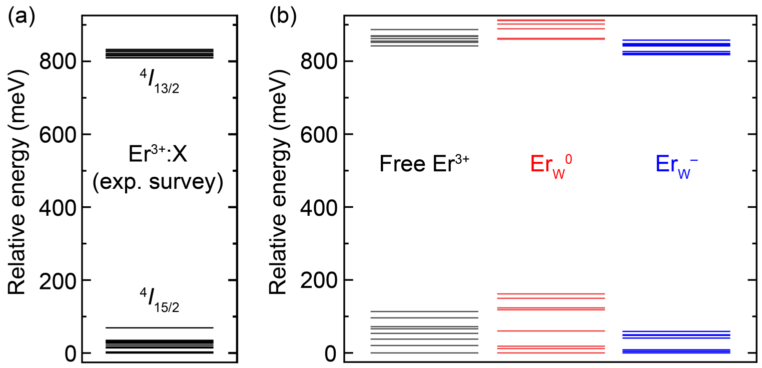

Fig. S9: Experimental and calculated many-body spectra. (a) An experimental survey of CF levels for Er in a variety of solid-state materials (Refs. 13 through 30). (b) Calculated many-body spectrum for the isolated Er^{3+} ion in free-space, and the Er_W defect in two different charge states.

II. Computational methods

a) Quantum embedding

The small spread in optical transitions observed in our experiments suggests a uniform distribution of defect configurations, activated via thermal annealing of the ion-implanted flakes. For this reason, we restrict our simulations to the substitutional Er defect in WS_2 (E_W), which is also found to be the most stable configuration for Er^{3+} ions in WS_2[31]. In order to estimate the optical spectrum of this system, we start by employing density functional theory (DFT) to calculate the relaxed atomic configuration and ground-state electronic structure of a single Er_W defect in monolayer WS_2. Due to strong electronic correlations in rare-earth ions, neglected at this level of theory, we use DFT only as a starting point to construct a semi-empirical effective model based on quantum embedding[32-34]. This approach allows us to capture many-body effects between strongly-localized defect states, providing a better description of the emerging optical properties[32-35].

Our effective model takes the form

$$\hat{H}_{eff} = \sum_{\langle ij \rangle} t_{ij} c_i^\dagger c_j + \frac{1}{2} \sum_{\langle ijkl \rangle} U_{ijkl}\, c_i^\dagger c_j^\dagger c_l c_k + \lambda \hat{L} \cdot \hat{S}\,, \tag{1}$$

where $c_i^\dagger$ (c) are creation (annihilation) operators, and i, j, k, l label Er $4f$ states, t_{ij} are hopping matrix elements, U_{ijkl} are screened Coulomb matrix elements, and λ is the strength of spin-orbit coupling whose operator is $\hat{L} \cdot \hat{S}$. The spin-orbit operator is constructed using the spin operators $\hat{S}_{x,y,z} = \frac{1}{2} \sum_{i\sigma\sigma'} c_{i\sigma}^\dagger \tau_{\sigma\sigma'}^{x,y,z} c_{i\sigma'}$ ($\tau^{x,y,z}$ are the Pauli matrices) and angular momentum operators $\hat{L}_{z,+,-} = \sum_{ii'\sigma} c_{i\sigma}^\dagger L_{ii'}^{z,+,-} c_{i'\sigma}$ where $L_{ii'}^z = i\delta_{i,i'}, L_{ii'}^+ = \delta_{i,i'+1}\sqrt{l(l+1) - i'(i'+1)},$ and $L_{ii'}^- = \delta_{i,i'+1}\sqrt{l(l+1) - i'(i'-1)}$, where we have used the basis of spherical harmonics ordered by increasing angular momentum quantum number.

We obtain the parameters of the effective model in the following way: The hopping matrix elements are determined from disentangling and Wannierizing the Er $4f$ manifold of states from (non-magnetic) DFT calculations[36] (details below). The direct output from the DFT is symmetrized under the D_{6h} point group. For U_{ijkl}, a spherically-symmetric Slater form is assumed with the Hunds coupling parameter J chosen such that for a calculation of an isolated Er^{3+}, the spectrum best matches the experiment[37-40]. A similar procedure is used to obtain λ, where only the quadratic part of the spin-orbit operator is retained.

We obtain the many-body states of the Er defect by diagonalizing Eq. (1), for which we use the Toolbox for Research on Interacting Quantum Systems (TRIQS) software library[41]. In Fig. S9, we compare the crystal-field (CF) levels for Er^{3+} in a variety of solid-state materials[13-30] with the many-body energy diagram obtained through our procedure for the isolated Er^{3+} ion, as well as the neutral and negatively charged Er_W

defects. The largest crystal-field (CF) splitting for the $^4I_{15/2}$ manifold of the Er_W^0 defect is ~180 meV (note that for the isolated Er^{3+}, the CF comes from periodic interactions and is thus arbitrary). For the Er_W^- case, we find a decrease in CF splitting, especially for the $^4I_{15/2}$ manifold. Although DFT is likely to overestimate CF interactions for this system[42], these results suggest that charge compensation around the Er_W defect has a strong impact on the overall CF splitting.

Lastly, we derive the Er_W optical absorption by evaluating the dipole matrix elements across the many-body spectrum using

$$\mu_{ij} = \langle \Psi_i | \hat{\boldsymbol{r}} | \Psi_j \rangle \,, \tag{2}$$

where $\Psi_{i,j}$ denote many-body wave functions of states i and j, and $\hat{\boldsymbol{r}}$ represents the many-body dipole operator expressed as

$$\hat{\boldsymbol{r}} = \sum_{nm} \boldsymbol{r}_{nm}^{wann}(\boldsymbol{0}) c_n^{\dagger} c_m \,. \tag{3}$$

The above expression uses the matrix elements of the position operator between Wannier functions m and n, given by

$$\boldsymbol{r}_{nm}^{Wann}(\boldsymbol{R}) = \langle n\boldsymbol{0} | \boldsymbol{r} | m\boldsymbol{R} \rangle. \tag{4}$$

This approach allows for transition dipole matrix elements between explicitly many-body ground and excited states of the Er_W defect, which is important to obtain quantitative transition amplitudes.

For convenience, we fold the optical matrix elements from Eq. (2) onto Lorentzian line-shapes to represent the absorption "strength function" spectrum, i.e.,

$$\tilde{\mu}_{ij} = \sum_{\langle ij \rangle} \frac{\mu_{ij}}{1 + 4\sigma^{-2}\left(E - E_{ij}\right)^2} \tag{5}$$

with arbitrary broadening factor $\sigma = 0.10$ meV chosen to resolve closely spaced transitions.

b) DFT, correlated active space, and computational details

All DFT calculations are performed using the VASP code[43], with exchange-correlation interactions described via the semi-local generalized-gradient approximation (GGA) parameterized by Pedrew, Burke and Ernzerhof (PBE)[44]. A kinetic energy cutoff of 700 eV is chosen for the plane-wave basis to ensure well-converged electronic structures and properly capture the highly-localized $4f$ electrons studied herein. We make use of projector-augmented wave (PAW) pseudopotentials to treat core electrons[45], with the Er $4f$ electrons treated explicitly within the valence. We model the defect by using a $7 \times 7 \times 1$ supercell containing a single Er_W substitution. The vacuum region included to avoid interlayer (van der Waals) interactions is 20 Å. All defect calculations are employed at the Γ-point only. We make use of tight convergence criteria for forces (0.001 eV/Å) and electronic iterations (10^{-8} eV) to ensure well-converged structures and wave functions. The ground-state atomic structure of the Er_W defect is obtained via spin-polarized DFT calculations, which we expect to be more realistic than that obtained without spin polarization (non-magnetic).

For all Wannier function calculations, we use the Wannier90 code[36] as interface with VASP. Since the experimental optical transitions are very close to those observed in other Er-doped systems, we assume these to be $4f \leftrightarrow 4f$ transitions and aim at studying the optical transitions only within the $4f$ manifolds of the proposed Er-related defect. Thus, we define the full $4f$ manifold (14 states) as the active space for our quantum embedding calculations, containing 11 electrons (three of which are unpaired) for the case of Er^{3+}. The single-particle occupation of Er^{3+} is found to remain unchanged in both the neutral and negatively charged states of Er_W. With this in mind, we define a disentanglement energy window of $[-3.15, +0.50]$ eV around the Fermi level[46] to capture important hybridization effects between the Er states and those from the bulk/dangling bonds.

References

[1] K. Wittmaack, “Reliability of a popular simulation code for predicting sputtering yields of solids and ranges of low-energy ions”, *J. Appl. Phys.* ***96*** *(2004) 2632.*

[2] P.L. Grande, F.C. Zawislak, D. Fink, M. Behar, “Range parameters study of medium-heavy ions implanted into light substrates”, Nucl. Instr. Meth, **61** (1991).

[3] S. Moll, Y. Zhang, Z. Zhu, P.D. Edmondson, F. Namavar, W.J. Weber, “Comparison between simulated and experimental Au-ion profiles implanted in nanocrystalline ceria”, *Nucl. Instr. Meth. Phys. Res. B* **307**, 93 (2013).

[4] Y. Zhang, I.T. Bae, K. Sun, C.M. Wang, M. Ishimaru, Z. Zhu, W. Jiang, W.J. Weber, “Damage profile and ion distribution of slow heavy ions in compounds”, *J. Appl. Phys.* ***105*** *(2009) 104901.*

[5] A. Polman, *“*Erbium implanted thin film photonic materials”, *J. Appl. Phys.* ***82***, 1–39 (1997).

[6] K. Eder, V. Bhatia, J. Qu, B. Van Leer, M. Dutka, J.M. Cairney, “A multi-ion plasma FIB study: Determining ion implantation depths of Xe, N, O and Ar in tungsten via atom probe tomography”, *Ultramicrosc*. **228**, 113334, 2021.

[7] C. Borschel, C. Ronning, “Ion beam irradiation of nanostructures A 3D Monte Carlo simulation code”, *Nucl. Instrum. Methods Phys. Res. B* **269**, 2133 (2011).

[8] W. Möller, “TRI3DYN, “Collisional computer simulation of the dynamic evolution of 3-dimensional nanostructures under ion irradiation””, *Nucl. Instr. Meth. Phys. Res. B* **322**, 23 (2014).

[9] S.O. Kucheyeva, J.E. Bradby, S. Ruffell, C.P. Li, T.E. Felter, A.V. Hamza, “Segregation and precipitation of Er in Ge”, *Appl. Phys. Lett.* **90**, 221901 (2007).

[10] N. Prtljaga, D. Navarro-Urrios, A. Tengattini, A. Anopchenko, J.M. Ramírez, J.M. Rebled, S. Estradé, J-P. Colonna, J-M. Fedeli, B. Garrido, L. Pavesi, “Limit to the erbium ions emission in silicon-rich oxide films by erbium ion clustering”, *Opt. Mater. Exp.* **2**, 1278 (2012).

[11] R.C. Powell, B. DiBartolo, B. Birang, C.S. Naiman, “Temperature dependence of the widths and positions of the r and n lines in heavily doped ruby”, *J. Appl. Phys.* **37**, 4973 (1966).

[12] B. Peng, H. Zhang, H. Shao, Y. Xu, X. Zhanga, H. Zhu, “Thermal conductivity of monolayer MoS_2, $MoSe_2$, and WS_2: Interplay of mass effect, interatomic bonding and anharmonicity”, *RSC Adv.* **6**, 5767 (2016).

[13] P. Stevenson, C.M. Phenicie, I. Gray, S.P. Horvath, S. Welinski, A.M. Ferrenti, A. Ferrier, P. Goldner, S. Das, R. Ramesh, R.J. Cava, N.P. de Leon, J.D. Thompson, “Erbium-implanted materials for quantum communication applications”, *Phys. Rev. B* **105***,* 224106 (2022).

[14] G.D. Grant, J. Zhang, I. Masiulionis, S. Chattaraj, K.E. Sautter, S.E. Sullivan, R. Chebrolu, Y. Liu, J.B. Martins, J. Niklas, A.M. Dibos, S. Kewalramani, J.W. Freeland, J. Wen, O.G. Poluektov, F.J. Heremans, D.D. Awschalom, S. Guha, “Optical and microstructural characterization of Er^{3+} doped epitaxial cerium oxide on silicon”, *APL Mater.* **12**, 021121 (2024).

[15] J. Zhang, G.D. Grant, I. Masiulionis, M.T. Solomon, J.C. Marcks, J.K. Bindra, J. Niklas, A.M. Dibos, O.G. Poluektov, F.J. Heremans, S. Guha, D.D. Awschalom, “Optical and spin coherence of Er spin qubits in epitaxial cerium dioxide on silicon”, *npj Quant. Inf.* **10**, 119 (2024).

[16] J. Bao, N. Yu, F. Capasso, “Controlled modification of erbium lifetime in silicon dioxide with metallic overlayers”, *Appl. Phys. Lett.* **91**, 131103 (2007).

[17] S-Y. Seo, J.H. Shin, C. Lee, “Long luminescence lifetime of 1.54 μm Er^{3+} luminescence from erbium doped silicon rich silicon oxide and its origin”, *MRS Online Proc. Lib.* **536**, 75 (1998).

[18] A. Gritsch, L. Weiss, J. Früh, S. Rinner, A. Reiserer “Narrow optical transitions in erbium-implanted silicon waveguides”, *Phys. Rev. X* **12**, 041009 (2022).

[19] Z. Tu, J. Zhang, J. Rönn, C. Alonso-Ramos, X. Leroux, L. Vivien, Z. Sun, É. Cassan, “Potential for sub-mm long erbium-doped composite silicon waveguide DFB lasers”*, Sci. Rep.* **10**, 10878 (2020).

[20] F. Priolo, G. Franzo, S. Coffa, A. Carnera, “Excitation and nonradiative deexcitation processes of Er3+ in crystalline Si”, *Phys. Rev. B* **57**, 4443 (1998).

[21] D.T.X Thao, C.A.J Ammerlaan, T. Gregokiewicz, “Photoluminescence of erbium-doped silicon: Excitation power and temperature dependence”, *J. Appl. Phys.* **88**, 1443 (2000).

[22] M.S. Bresler, O.B. Gusev, E.I. Terukov, I.N. Yassievich, B.P. Zakharchenya, V.I. Emel'yanov, B.V. Kamenev, P.K. Kashkarov, E.A. Konstantinova, V.Yu. Timoshenko, "Stimulated emission in erbium-doped silicon structures under optical pumping", *Mater. Sci. Eng. B* **81**, 52 (2001).

[23] A.J. Kenyon, "Erbium in silicon", *Semicond. Sci. Technol*. **20**, R65 (2005).

[24] H.A. Lopez, P.M. Fauchet, "Infrared LEDs and microcavities based on erbium-doped silicon nanocomposites", *Mater. Sci. Eng. B* **81**, 91 (2001).

[25] G. Mula, T. Printemps, C. Licitra, E. Sogne, F. D'Acapito, N. Gambacorti, N. Sestu, M. Saba, E. Pinna, D. Chiriu, P.C. Ricci, A. Casu, F. Quochi, A. Mura, G. Bongiovanni, A. Falqui, "Doping porous silicon with erbium: pores filling as a method to limit the Er-clustering effects and increasing its light emission", *Sci. Rep.* **7**, 5957 (2017).

[26] C. Yin, M. Rancic, G.G. de Boo, N. Stavrias, J.C. McCallum, M.J. Sellars, S. Rogge, "Optical addressing of an individual erbium ion in silicon", *Nature* **497**, 91 (2013).

[27] W. Redjem, A. Durand, T. Herzig, A. Benali, S. Pezzagna, J. Meijer, A. Yu. Kuznetsov, H.S. Nguyen, S. Cueff, J.-M. Gérard, I. Robert-Philip, B. Gil, D. Caliste, P. Pochet, M. Abbarchi, V. Jacques, A. Dréau, G. Cassabois, "Single artificial atoms in silicon emitting at telecom wavelengths", *Nat. Electr. Rev.* **3**, 738 (2020).

[28] T. Böttger, C.W. Thiel, Y. Sun, R.L. Cone, "Optical decoherence and spectral diffusion at 1.5 μm in Er^{3+}:Y_2SiO_5 versus magnetic field, temperature, and Er^{3+} concentration"*, Phys. Rev. B* **73**, 075101 (2006).

[29] F. Zhang, Z. Bi, J. Chen, A. Huang, Y. Zhu, B. Chen, Z. Xiao "Spectroscopic investigation of Er^{3+} in fluorotellurite glasses for 2.7 μm luminescence", *J. Alloys Comp.* **649**, 1191 (2015).

[30] F. Chen, T. Wei, X. Jing, Y. Tian, J. Zhang, S. Xu, "Investigation of mid-infrared emission characteristics and energy transfer dynamics in Er3+ doped oxyfluoride tellurite glass", *Sci. Rep.* **5**, 10676 (2015).

[31] G.I. López-Morales, A. Hampel, G.E. López, V.M. Menon, J. Flick, C.A. Meriles, "Ab-initio investigation of Er^{3+} defects in tungsten disulfide", *Comp. Mater. Sci.* **210**, 111041 (2022).

[32] M. Bockstedte, F. Schütz, T. Garratt, V. Ivády, A. Gali, "Ab initio description of highly correlated statesin defects for realizing quantum bits", *npj Quant. Mater.* **3**, 31 (2018).

[33] H. Ma, N. Sheng, M. Govoni, G. Galli, "Quantum embedding theory for strongly correlated states in materials", *J. Chem. Theor. Comp.* **17**, 2116 (2021).

[34] L. Muechler, D.I. Badrtdinov, A. Hampel, J. Cano, M. Rösner, C.E. Dreyer, "Quantum embedding methods for correlated excited states of point defects: Case studies and challenges", *Phys. Rev. B* **105**, 235104 (2022).

[35] G.I. López-Morales, J.M. Zajac, J. Flick, C.A. Meriles, C.E. Dreyer, "Quantum embedding study of strain and charge induced stark effects on the NV− center in diamond", *Phys. Rev. B*, **110**, 245127 (2024).

[36] G. Pizzi, V. Vitale, R. Arita, S. Blügel, F. Freimuth, G. Géranton, M. Gibertini, D. Gresch, C. Johnson, T. Koretsune, J. Ibañez-Azpiroz, H. Lee, J.-M. Lihm, D. Marchand, A. Marrazzo, Y. Mokrousov, J.I. Mustafa, Y. Nohara, Y. Nomura, L. Paulatto, S. Poncé, T. Ponweiser, J. Qiao, F. Thöle, S. S. Tsirkin, M. Wierzbowska, N. Marzari, D. Vanderbilt, I. Souza, A.A. Mostofi, J.R. Yates, "Wannier90 as a community code: new features and applications", *J. Phys.: Cond. Matter* **32**, 165902 (2020).

[37] T. Schweizer, D.J. Brady, D.W. Hewak, "Fabrication and spectroscopy of erbium doped gallium lanthanum sulphide glass fibres for mid-infrared laser applications", *Opt. Exp.* **1**, 102 (1997).

[38] T. Wei, Y. Tian, C. Tian, X. Jing, J. Zhang, L. Zhang, S. Xu, "Optical spectroscopy and population behavior between 4I11/2 and 4I13/2 levels of erbium doped germanate glass", *Opt. Mater. Exp.* **4**, 2150 (2014).

[39] M.F. Reid, "Theory of rare-earth electronic structure and spectroscopy", *Handbook Phys. Chem. Rare Earths* **50**, 47 (2016).

[40] B. Hou, M. Jia, P. Li, G. Liu, Z. Sun, Z. Fu, "Multifunctional optical thermometry based on the rare-earth-ions-doped up-/down-conversion Ba2TiGe2O8: Ln (Ln =Eu3+/ Er3+/ Ho3+/ Yb3+) phosphors", *Inorg. Chem.* **58**, 7939 (2019).

[41] O. Parcollet, M. Ferrero, T. Ayral, H. Hafermann, I. Krivenko, L. Messio, P. Seth, "Triqs: a toolbox for research on interacting quantum systems", *Comp. Phys. Comm.* **196**, 398 (2015).

[42] Y. Limbu, Y. Shi, J. Sink, T.O. Puel, D. Paudyal, M.E. Flatté, "Ab initio calculations of erbium crystal field splittings in oxide hosts", *arXiv*, arXiv:2501.03348 (2025).

[43] G. Kresse, J. Furthmüller, "Efficient iterative schemes for ab initio total-energy calculations using a plane-wave basis set", *Phys. Rev. B* **54**, 11169 (1996).

[44] J. P. Perdew, K. Burke, and M. Ernzerhof, "Generalized gradient approximation made simple", *Phys. Rev. Lett.* **77**, 3865 (1996), erratum *Phys. Rev. Lett.* **78**, 1396 (1997).

[45] P. E. Blöchl, "Generalized gradient approximation made simple", *Phys. Rev. B* **50**, 17953 (1994).

[46] I. Souza, N. Marzari, D. Vanderbilt, "Maximally localized Wannier functions for entangled energy bands", *Phys. Rev. B* **65**, 035109 (2001).
